# White matter microstructure of patients with neurofibromatosis type 1 and its relation to inhibitory control

**DOI:** 10.1007/s11682-016-9641-3

**Published:** 2016-10-29

**Authors:** M. Koini, S. A. R. B. Rombouts, I. M. Veer, M. A. Van Buchem, S. C. J. Huijbregts

**Affiliations:** 1 Institute of Psychology, Leiden University, Leiden, The Netherlands; 2 Leiden Institute for Brain and Cognition (LIBC), Leiden University, Leiden, The Netherlands; 3 Department of Neurology, Medical University of Graz, Auenbruggerplatz 22, A-8036 Graz, Austria; 4 Department of Radiology, Leiden University Medical Center, Leiden, The Netherlands; 5 Department of Psychiatry and Psychotherapy, Division of Mind and Brain Research, Charité Universitaetsmedizin Berlin, Berlin, Germany; 6 Department of Clinical Child and Adolescent Studies, Leiden University, Leiden, The Netherlands

**Keywords:** Neurofibromatosis type I, DTI, Anterior thalamic radiation, Executive functions

## Abstract

Neurofibromatosis Type 1 (NF1) is commonly associated with deficits in executive functions such as working memory and inhibitory control. A valid biomarker to describe the pathological basis of these deficits in NF1 is not available. The aim of this study was to investigate whether any abnormalities in white matter integrity of the executive function related anterior thalamic radiation (ATR), cingulate bundle (CB), and superior longitudinal fasciculus (SLF) may be regarded as a pathological basis for inhibitory control deficits in adolescents with NF1. Sixteen NF1 patients and 32 healthy controls underwent 3 T DTI MRI scanning. Whole brain-, ATR-, CB-, and SLF-white matter integrity were studied using fractional anisotropy, mean (MD), radial, and axial (DA) diffusivity. Correlation analyses between white matter metrics and inhibitory control (as measured with a computerized task) were performed. Also, verbal and performance abilities (IQ-estimates) were assessed and correlated with white matter metrics. Patients showed significant whole brain- and local microstructural pathology when compared to healthy controls in all measures. In NF1-patients, whole-brain (MD: *r* = .646 and DA: *r* = .673) and ATR- (r-range: −.405–.771), but not the CB- (r-range: −.307–.472) and SLF- (r-range: −.187–.406) white matter integrity, were correlated with inhibitory control. Verbal and performance abilities were not associated with white matter pathology. In NF1, white matter abnormalities are observed throughout the brain, but damage to the ATR seems specifically, or at least most strongly related to inhibitory control. Future studies should examine whether reduced white matter integrity in other brain regions or tracts is (more strongly) associated with different aspects of the cognitive-behavioral phenotype associated with NF1.

## Introduction

Neurofibromatosis Type 1 (NF1) is a single-gene disorder affecting approximately 1 in 3.500 individuals (National Institutes of Health. 1988). In NF1, a loss of function mutation of the neurofibromin gene leads to increased expression of rat sarcoma (Ras-) proteins (name based on discovery of two cancer-causing viruses in rats). Ras is expressed in all cell lineages and organs (including the brain), and both directly and indirectly, through for instance the MAPK- and PI_3_K/AKT/mTOR pathways, regulates intracellular signaling. Loss of neurofibromin and subsequent increased Ras-activity have been associated with abnormalities in (neural) cell differentiation, growth, and apoptosis (Dasgupta and Gutmann 2005; National Institutes of Health. 1988; Shilyansky et al. 2010; Tidyman and Rauen 2009).

In line with the pathophysiological loss of control over these cellular processes, including neuronal cells, brain imaging studies have shown various cerebral abnormalities in NF1. These include volumetric abnormalities (mainly increased white matter and subcortical gray matter volumes, and decreased cortical grey matter density) (S. C. Huijbregts et al. 2015) and lower cortical gyrification (Violante et al. 2013). Other frequently-observed abnormalities are focal areas of hyperintensities on T2-weighted images, of which the exact nature is not yet known (Billiet et al. 2014) and reduced integrity of white matter microstructure (Ferraz-Filho et al. 2011; Karlsgodt et al. 2012; van Engelen et al. 2008). Damage to white matter microstructure was found in the entire brain (Karlsgodt et al. 2012).

NF1-patients also exhibit very high rates of cognitive and social impairment. Specifically, 60–80 % of children with NF1 experience (specific) learning disabilities (Hyman et al. 2005), up to 60 % show mild to severe autism symptoms, and up to 50 % qualifies for a diagnosis of Attention Deficit (Hyperactivity) Disorder (Garg et al. 2013; Hyman et al. 2005). Considering cognition, executive dysfunction is a major hallmark of the disease (Diggs-Andrews and Gutmann 2013; Huijbregts et al. 2010b; Rowbotham et al. 2009). Core executive functions include inhibitory control, working memory and cognitive flexibility (Miyake et al. 2000). All of these are affected in NF1 (Huijbregts et al. 2010b; Rowbotham et al. 2009). For executive functioning, positive associations have been reported between executive impairment and subcortical volumes in NF1 (S. C. Huijbregts et al. 2015). With respect to the impact of T2-hyperintensities on cognition assocations with lesion location have been revealed, but not with presence or number (Hyman et al. 2007; Moore et al. 1996).

Surprisingly, no studies have been performed yet examining associations between white matter microstructural abnomalities and the cognitive-behavioral phenotype of NF1, although it has been suggested that presence of T2-hyperintensities might impair functioning of white matter tracts and subsequently cognition (Payne et al. 2014). White matter integrity may be the best neural substrate for functional connectivity, which several recent studies have shown to be abnormal in NF1 (Loitfelder et al. 2015; Tomson et al. 2015). Also, abnormalities in functional connectivity have been related to parent-reported problems with executive functioning in NF1 (Loitfelder et al. 2015).

Reduced microstructural white matter integrity in adult NF1 patients compared to healthy controls was reported to be present in the entire brain, but most prominently in the anterior thalamic radiation (ATR) (Karlsgodt et al. 2012). The ATR connects the thalamus with the frontal cortex and its strategic role in executive functioning has been shown in other disorders, such as CADASIL (Duering et al. 2011), a hereditary stroke disorder, schizophrenia (Mamah et al. 2010), and first episode psychosis (Pérez-Iglesias et al. 2010). Although not specifically highlighted in NF1, other tracts such as the cingulate bundle, projecting from the posterior cingulate cortex to the medial prefrontal cortex (Gordon et al. 2011), and the superior longitudinal fasciculus, which connects the parietal, occipital and temporal lobes with ipsilateral frontal cortices (Schmahmann et al. 2008) have also been associated with executive functioning (Heilbronner and Haber 2014; Mesulam 1998; Nestor et al. 2004; Petrides and Pandya 2002).

In the present study, it was investigated whether whole brain white matter integrity and white matter integrity in tracts specifically associated with executive functions were related to inhibitory control in adolescents with NF1.

## Methods

### Participants

Sixteen NF1 patients (7 male, age: M = 12.45, SD = 2.75, min-max: 9.3–18.6) and 32 healthy controls (21 male, age: M = 12.43, SD = 2.99, min-max: 9.2–19.0; t_age_ = −0.014, p_age_ = .989; χ^2^_sex_ = 1.835, p_sex_ = .176) underwent structural MRI scanning. All NF1 subjects fulfilled the diagnostic criteria specified by the National Institutes of Health Consensus Conference (National Institutes of Health. 1988).

### Scanning procedure

All subjects underwent scanning at the Leiden University Medical Center. Imaging was performed on a Philips 3 Tesla Achieva MRI scanner using an 8 channel SENSE receiver head coil (Philips Healthcare, Best, The Netherlands). In each subject, DTI data were acquired using a 16 directions spin echo sequence with 73 slices without a gap in AC-PC direction (TR 8.36 s, TE 56 ms, flip angle 90 degrees, 2.3 mm isotropic voxels, FOV = 220 × 167.9, b-value 1000s/mm2, total scan time of 183 s, and one B0-image). For the evaluation of T2-hyperintensities, T2-weighted structural scans were acquired (52 slices, voxel size: 0.43 × 0.478, 3 mm slice thickness, FOV: 220x175x156, TE 80 ms, no slice gap, scan duration 236 s). All anatomical scans were reviewed by a neuro-radiologist.

### Image processing

All analyses were performed using FMRIB’s Software Library (Smith et al. 2004). Preprocessing included pre-alignment, correction for eddy currents, and brain extraction. FMRIB’s Diffusion Toolbox was used to reconstruct diffusion tensors, fitting a diffusion tensor model at each voxel. Tensor eigenvalues describing the diffusion strength in primary, secondary and tertiary diffusion directions were extracted. Voxelwise statistical analysis of the fractional anisotropy (FA) data was carried out using Tract-Based Spatial Statistics (TBSS (Smith et al. 2006)). All subjects’ FA data were aligned into a common space using the nonlinear registration tool (FMRIB’s non-linear registration tool, (Smith et al. 2004)). Next, the mean FA image was created and thinned to create a mean FA skeleton which represents the centers of all tracts common to the group. Each subject’s aligned FA data was then projected onto this skeleton and the resulting data fed into voxelwise cross-subject statistics. FA, mean diffusivity (MD), axial diffusivity (DA) and radial diffusivity (RD) were used as measures of microstructural white matter integrity. RD was calculated using the average of the secondary and the tertiary eigenvectors (L2 + L3/2). Each additional measure (MD, DA, and RD) was projected on the FA-skeleton. Permutation-based inferences (FSL’s randomize, 5000 permutations, FWE-corrected, *p* = 0.05) with threshold-free cluster enhancement (TFCE) were carried out for voxelwise analyses of the skeleton. Two sample t-tests were used for the whole brain voxelwise group comparisons (NF1 patients vs. healthy controls).

**Fig. 1:**
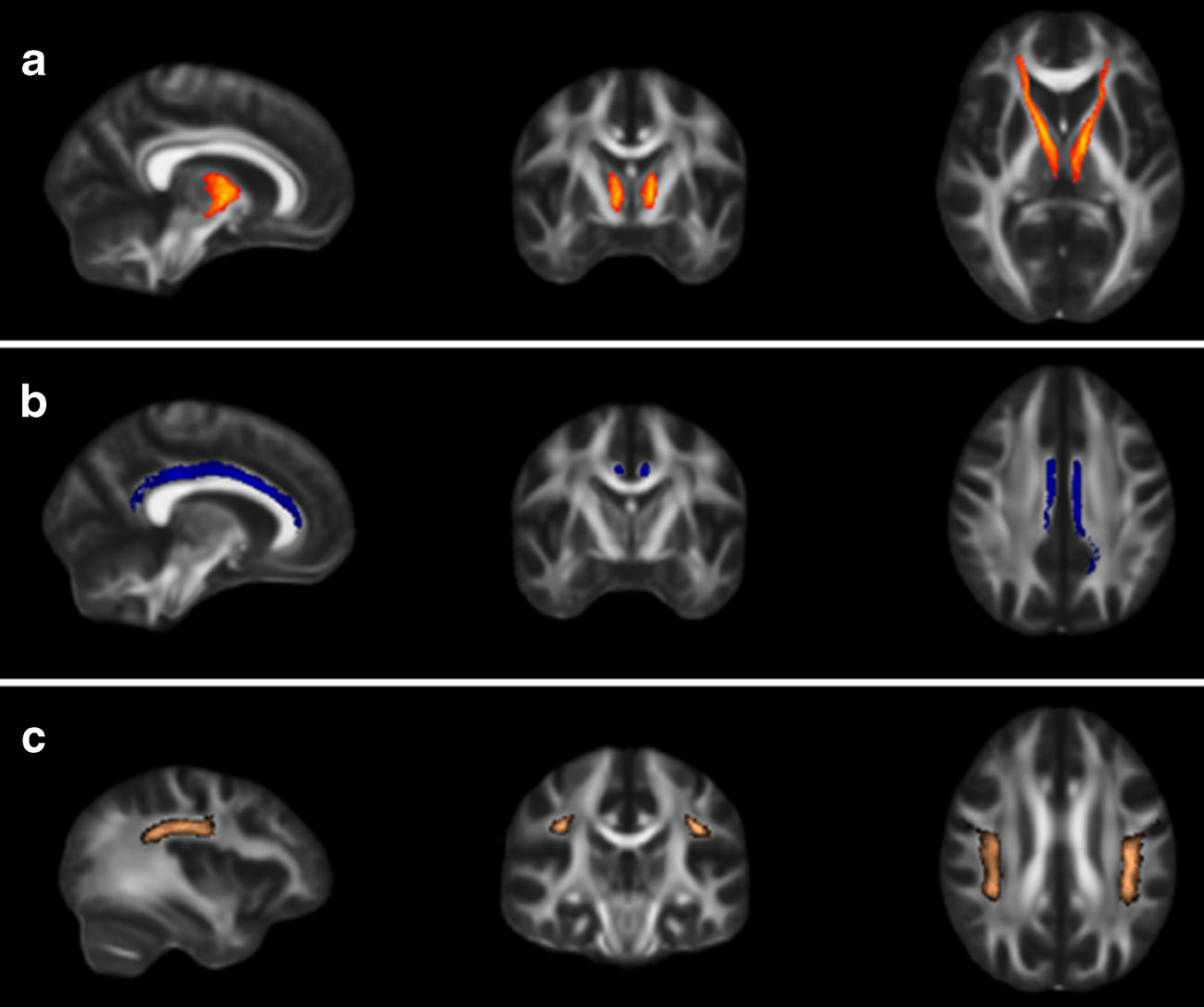
Schematic illustration of the anterior thalamic radiation (ATR, a), the cingulate bundle (CB, b) and the superior longitudinal fasciculus (SLF, c)

Thereafter, masks of the ATR, the cingulate bundle (CB) and the superior longitudinal fasciculus (SLF) (Fig. 1) were created using binarised regions of interest, which were based on the John Hopkins University White-Matter Tractography Atlas (Mori et al. 2005). To ensure value extraction in white matter only, the mask was applied to the mean FA skeleton. This confines the statistical analysis exclusively to voxels from the center of the tract, thereby minimizing anatomic inter-subject variability, registration errors, and partial volume effects. Averaged FA, MD, DA, and RD values of the regions of interest were used to examine group differences and the relation with inhibitory control.

### Neuropsychological assessment

The Sustained Attention Dots-task from the computerized Amsterdam Neuropsychological Tasks (ANT) was used to evaluate inhibitory control (S C J Huijbregts et al. 2002). In the Sustained Attention Dots-task (600 trials) participants press the yes-button (i.e. a response with the index finger of the dominant hand) when 4 randomly placed dots appear on the computer screen (200 trials), and the no-button (a response with the index finger of the non-dominant hand) when 3 or 5 randomly placed dots appear (total of 400 trials). As pressing the no-button becomes the predominant (“automatic”) response, inhibitory control is required when responses with the yes-button have to be given. Lack of accuracy of task performance (error rate) was used to represent quality of inhibitory control. Neuropsychological assessment also included the following subtests from the Wechsler Intelligence Scale for Children (Wechsler et al. 2011): Vocabulary and Comprehension (verbal comprehension), Block-Design (visual-spatial abilities), Picture Completion (fluid reasoning), Symbol Search, and Coding (processing speed). Verbal abilities were estimated based on the z-transformed subscales vocabulary and comprehension, performance abilities were estimated on the basis of all other z-transformed subtests. Neuropsychological data were collected for 13 NF1 patients and 9 HC.

### Statistical analyses

Statistical analyses were performed using SPSS 21 for Windows (SPSS, Chicago, IL). Whenever appropriate, non-parametric procedures were applied. Correlation analyses between averaged whole brain or averaged tract-based DTI metrics, and inhibitory control, and verbal and performance abilities were conducted (false discovery rate corrected for multiple comparisons, q = .05). Correlation analyses were conducted in patients only. To increase accuracy of sampling estimates, 1000 equally sized random samples with replacement were generated (bootstrapping) using bias corrected and accelerated intervals. The confidence intervals of the bootstrapping approach were used for inferences. Also, differences in DTI-metrics between left and right hemisphere were assessed. To indicate strength of results, relative deviation in white matter microstructure compared to healthy controls, effect size (Cohen’s d) and percent of non-overlap of the group specific distributions were calculated.

### T2 hyperintensities

The T2 scans from NF1 patients were visually checked by a neuroradiologist for presence of T2-hyperintensities. Next, hand-labelled masks were created of all voxels showing T2-hyperintensities. To examine the potential influence of thalamic T2-hyperintensities (for which specifically effects on cognition had been shown: (Hyman et al. 2007; Moore et al. 1996; Payne et al. 2014)) on the ATR-microstructure, non-parametric group comparisons using the ATR’s FA, MD,RD, and DA between patients with and without T2-hyperintensities were conducted.

## Results

### Voxel-wise microstructural analysis of the whole brain

**Fig. 2:**
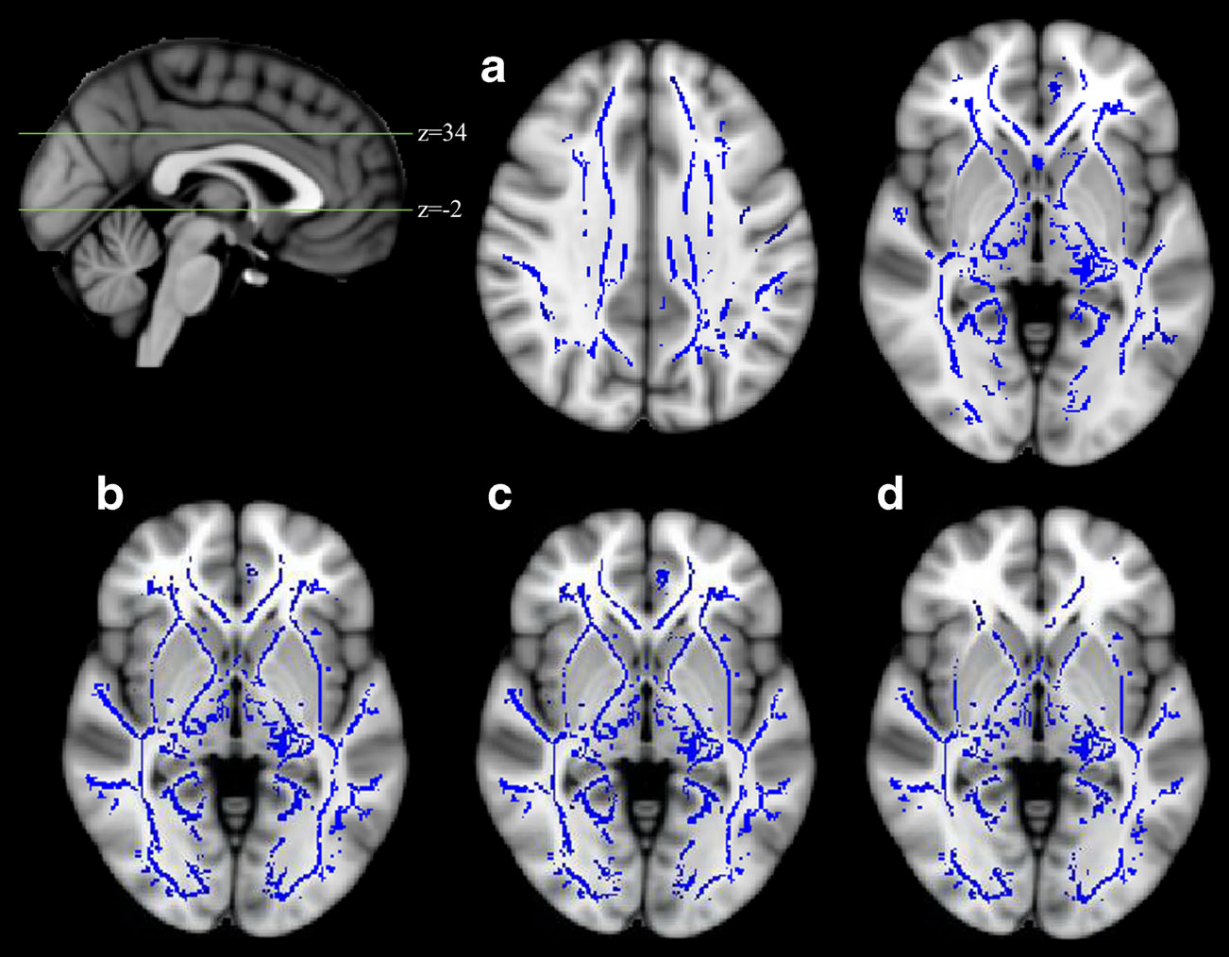
Whole brain group differences in fractional anisotropy (a, NF1 patients < controls), mean diffusivity (b, NF1 patients > controls), radial diffusivity (c, NF1 patients > controls) and axial diffusivity (d, NF1 patients > controls), TFCE and FWE corrected, *p* < 0.05

**Fig. 3:**
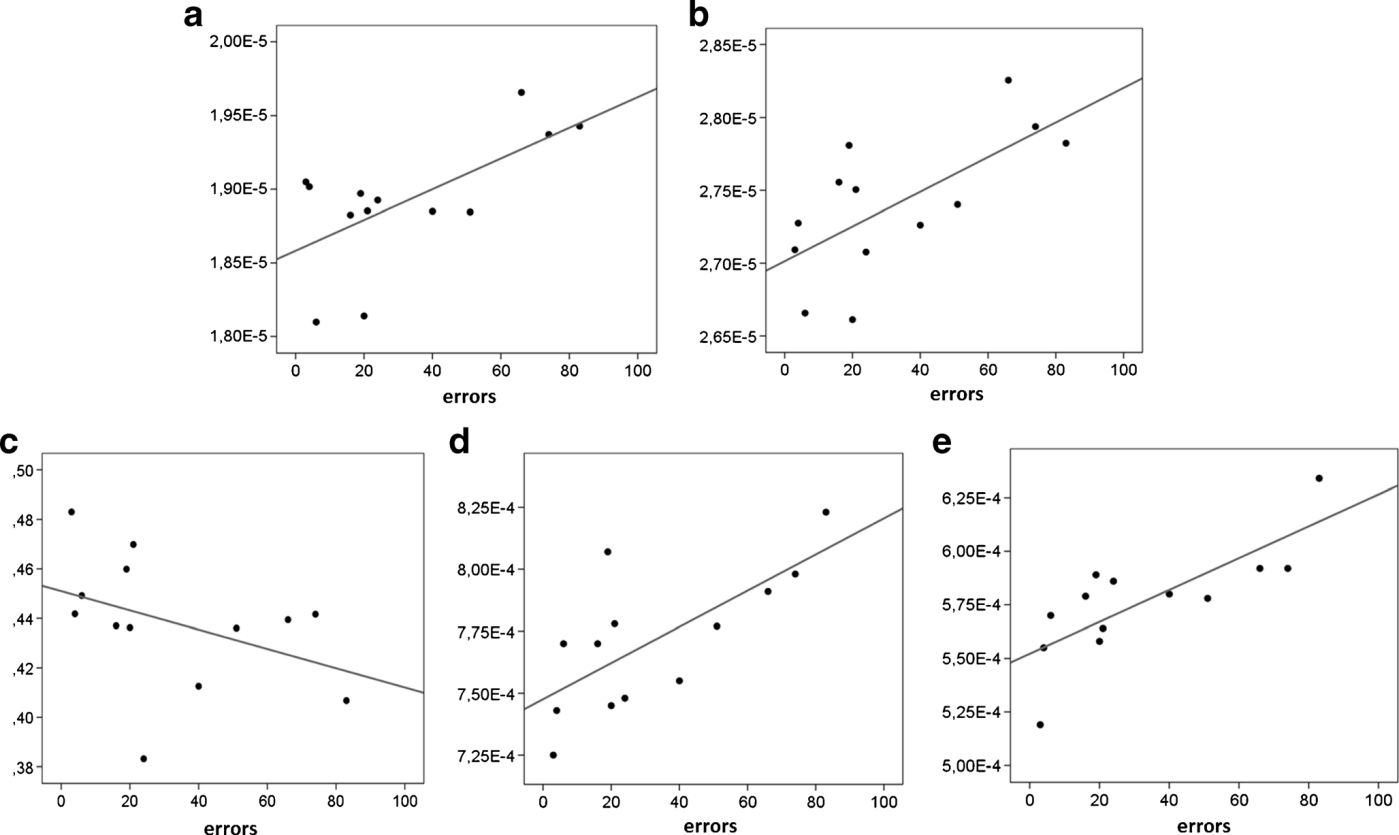
Scatter plots showing errors in inhibitory control and whole brain mean diffusivity (**a**), whole brain axial diffusivity (**b**), fractional anisotropy of left ATR (**c**), mean diffusivity of left ATR (**d**) , and radial diffusivity of the left ATR (**e**)

Whole brain voxel-wise permutation analyses revealed a reduction of FA and an increase of MD, RD, and DA throughout the whole brain in NF1 adolescents when compared to controls (Fig. 2). Correlation analyses in NF1 patients revealed an association between the averaged values of the MD and inhibitory control (*r* = .646, CI_bootstrap_ = 0.034–0.882), and the averaged values of the DA and inhibitory control (*r* = .673, CI_bootstrap_ = 0.272–0.861) (Fig. 3). No significant correlation was identified for the averaged values of the whole brain DTI metrics and our estimates of verbal, performance and total abilities.

### The microstructure of cingulate bundle-, superior longitudinal fasciculus-, and ATR-white matter

**Fig. 4:**
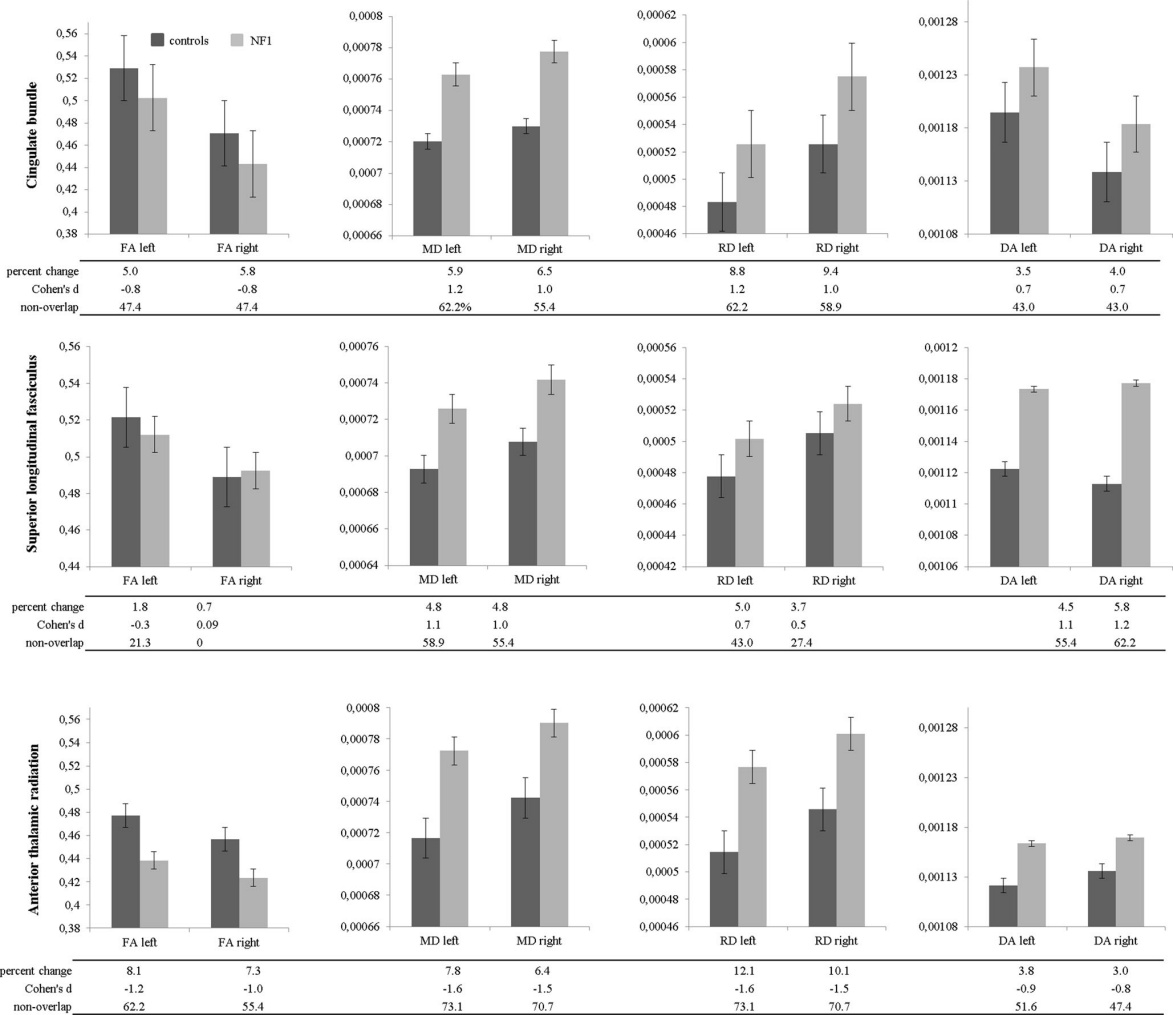
Mean DTI values (fractional anisotropy (FA), mean diffusivity (MD), radial diffusivity (RD), and axial diffusivity (DA)) of NF1 patients and healthy controls in the cingulate bundle, the superior longitudinal fasciculus, and the anterior thalamic radiation left and right. Cohen’s d and non-overlap provides information about the effect size and the non-overlapping distribution of patients and controls, respectively

Group comparisons using the averaged values of the cingulate bundle, the superior longitudinal fasciculus, and the ATR revealed for all but a few significantly decreased FA and increased MD, RD and DA in NF1 patients when compared to controls (Table 1). The averaged values of the FA, MD, RD, and DA of the CB, the SLF and the ATR showed differences between patients and controls in the range of 0.7 to 12.1 %, being most pronounced in the RD (Fig. 4). Also, large effect sizes (CB range: 0.7–1.2; SLF range: −0.3-1.2; ATR range: −0.8-(1.6)), which were associated with a high percentage of non-overlapping distributions were found (Fig. 4). To examine hemispherical differences in microstructural damage between left and right averaged CB, paired t-tests restricted to NF1 patients revealed differences in the FA (t(15) = 11.091, *p* < 0.001), RD (t(15) = −5.856, *p* < 0.001), and DA (t(15) = 3.162, *p* = 0.006), but not for MD (t(15) = −1.441, *p* = .170). In the SLF, FA (t(15) = 3.196, *p* = 0.006), MD (t(15) = −3.037, *p* = 0.008), and RD (t(15) = −3.738, *p* = 0.002), but not DA (t(15) = −0.394, *p* = 0.699) showed hemispherical differences. In the ATR, the FA (t(15) = 3.029, *p* = .008), MD (t(15) = −3.067, *p* = .008), and RD (t(15) = −.846, *p* = .002), but not the DA (t(15) = −.702, *p* = .494 ) showed significant differences between left and right hemisphere. All tracts indicate less microstructural damage to the left hemisphere, with the exception of the DA of the cingulate bundle. A similar effect was found in control subjects (CB: FA t(31) = 19.849, *p* < 0.001; MD t(31) = −2.134, *p* = 0.041; RD t(31) = −10.284, *p* < 0.001, DA t(31) = 7.867, *p* < 0.001; SLF: FA t(31) = 5.871, *p* < 0.001; MD t(31) = −4.385, *p* < 0.001; RD t(31) = −5.388, *p* < 0.001; DA t(31) = 1.572, *p* = 0.126; ATR: FA: t(31) = 5.832, *p* < 0.001; MD: U = −4.938, *p* < 0.001; RD: t(31) = −10.413, *p* < 0.001; DA: U = −3.009, *p* = 0.003).

**Table 1:** Differences between NF1 patients and controls in fractional anisotropy (FA), mean diffusivity (MD), radial diffusivity (RD), and axial diffusivity (DA) within the cingulate bundle, the superior longitudinal fasciculus, and the anterior thalamic radiation

		left			right		
		df	t	*p*	df	t	*p*
Cingulate bundle	FA	46	2.746	0.009	46	2.983	0.005
	MD	46	-4.623	<0.001	46	-3.856	<0.001
	RD	46	-4.755	<0.001	46	-4.199	<0.001
	DA	46	-2.241	0.030	46	-2.422	0.019
Superior longitudinal fasciculus	FA	46	0.995	0.325	46	-0.294	0.770
	MD	*	75.000	<0.001	*	100.500	0.001
	RD	46	-2.467	0.017	*	188.500	0.140
	DA	46	-3.972	<0.001	*	86.500	<0.001
Anterior thalamic radiation	FA	46	4.942	<0.001	46	4.111	<0.001
	MD	*	21.000	<0.001	*	35.500	<0.001
	RD	*	23.000	<0.001	46	-7.307	<0.001
	DA	46	-3.603	0.001	46	-3.058	0.004

*U-Test

### Relation of the cingulate bundle, the superior longitudinal fasciculus and the ATR with inhibitory control

No DTI-measure of the CB or the SLF revealed a significant correlation with our measure of inhibitory control in NF1 patients (CB: FA left: *r* = −.203, CI_bootstrap_ = −.785–.424; FA right: *r* = −.307, CI_bootstrap_ = −.776–.349; MD left: *r* = .350, CI_bootstrap_ = .-483–.879; MD right: *r* = −.009, CI_bootstrap_ = −.769–.653; RD left: *r* = .472, CI_bootstrap_ = −.366–.823; RD right: *r* = .099, CI_bootstrap_ = −.659–.709; DA left: *r* = .093, CI_bootstrap_ = −.477–.731; DA right: *r* = −.131, CI_bootstrap_ = −.778–.595; SLF: FA left: *r* = .055, CI_bootstrap_ = −.583–.720; FA right: *r* = −.187, CI_bootstrap_ = −.725–.483; MD left: *r* = .241, CI_bootstrap_ = .-512–.712; MD right: *r* = .406, CI_bootstrap_ = −.594–.902; RD left: *r* = .111, CI_bootstrap_ = −.643–.583; RD right: *r* = .331, CI_bootstrap_ = −.456–.790; DA left: *r* = .280, CI_bootstrap_ = −.387–.785; DA right: *r* = .347, CI_bootstrap_ = −.286–.772). In contrast, for the ATR associations with inhibitory control were evident for FA (*r* = −.405, CI_bootstrap_ = −0.752-(−0.060); not significant after correction for multiple comparisons), MD (*r* = .707, CI_bootstrap_ = 0.196–0.930), and RD (*r* = .771, CI_bootstrap_ = 0.490–0.919), all in the left hemisphere (Fig. 3). No significant correlations between FA, MD, RD, and DA of the CB, the SLF or the ATR and our IQ-estimates were found.

### Neuropsychological results

When compared to controls, NF1 patients showed significantly lower scores on our measure of performance abilities (M_HC_ = 0.606, SD_HC_ = 0.638; M_NF1_ = −0.474, SD_NF1_ = 0.812; t(18) = 3.250, *p* = .004) and total abilities (M_HC_ = 0.551, SD_HC_ = 0.620; M_NF1_ = −0.402, SD_NF1_ = 0.730; t(18) = 3.107, *p* = .006). A trend was found for verbal abilities (M_HC_ = 0.442, SD_HC_ = 0.680; M_NF1_ = −0.259, SD_NF1_ = 0.899; t(18) = 1.928, *p* = .070). Despite relatively large absolute differences in error rate, no significant group differences were found for inhibitory control between patients and controls (M_controls_ = 20.11 ± 15.36, M_NF1_ = 32.85 ± 27.37, t(20) = −1.259, *p* = .222).

### T2-hyperintensities

T2-hyperintensities were identified in 62.5 % (*n* = 10 of 16) of the patients, of which 31.3 % (*n* = 5) showed T2-hyperintensities in the thalamus, 37.5 % (*n* = 6) in the cerebellum, 25 % (*n* = 4) in the pallidum, 18.8 % (*n* = 3) in the brainstem and in the cortical grey matter, 6.3 % (*n* = 1) in the putamen and amygdala, and 31.3 % (*n* = 5) in the cerebral white matter. Also, 12.5 % (*n* = 2) subjects had one T2- hyperintensity in the left ATR. To determine whether thalamic T2-hyperintensities have an effect on the microstructural integrity of ATR, subjects were separated in two groups, thalamic T2-hyperintensities present (n = 5) and absent (*n* = 11). Non-parametric group comparisons revealed no significant differences in the ATR FA (left: *p* = 0.267, right: *p* = 0.377), MD (left: *p* = 0.320, right; *p* = 0.913), RD (left; *p* = 0.743, right: *p* = 0.913) or DA (left; *p* = 0.115, right: *p* = 0.267).

## Discussion

In this study we found extensive global and local white matter abnormalities in NF1 adolescents. The significance of white matter pathology and its impact on cognitive functioning has already been recognized in various neurological and neurodevelopmental disorders (Duering et al. 2011; Mamah et al. 2010; Pérez-Iglesias et al. 2010; Yu et al. 2012). In NF1, white matter pathology has also been observed before (Ferraz-Filho et al. 2011; Karlsgodt et al. 2012; van Engelen et al. 2008), but its functional significance as a biomarker for the quantification of impaired executive functioning had not yet been investigated. We found a number of associations between whole brain white matter integrity and inhibitory control, one of the core executive functions, in NF1. In order to achieve more specific results, we examined white matter integrity in three tracts, the cingulate bundle, the superior longitudinal fasciculus, and the anterior thalamic radiation, which have all been associated with executive functioning (Duering et al. 2011; Gordon et al. 2011; Heilbronner and Haber 2014; Mamah et al. 2010; Nestor et al. 2004; Pérez-Iglesias et al. 2010; Petrides and Pandya 2002; Schmahmann et al. 2008). Whereas indeed we found strong associations between white matter integrity of the ATR and inhibitory control, no significant associations were observed for the CB and the SLF. A possible explanation for the apparent specificity of the association between ATR WM-integrity and inhibitory control in this study might lie in the nature of the task that was used, or, in other words, in the type of inhibitory control measured by the Sustained Attention Dots-task -task. Inhibitory control as measured by the Sustained Attention Dots-task may be classified as “cool” executive functioning: it is “decontextualized”, and does not involve affect or motivation (Griffith-Lendering et al. 2012; Stephan C J Huijbregts et al. 2008; Rubia 2011). The ATR connects the thalamus and the frontal cortex and does not seem to involve projections from/to cortical or subcortical brain regions that have specifically been related to affect/emotion and motivation, whereas the CB connects brain regions associated with executive functions, decision-making and emotion (Gordon et al. 2011). The CB might therefore be associated more strongly with so-called “hot” executive functioning, i.e. executive functioning with an affective or motivational component or within an affective or motivational context (Griffith-Lendering et al. 2012; Stephan C J Huijbregts et al. 2008; Rubia 2011). Alternatively, however, the CB is one of the brain’s major white matter pathways and its specific architecture may have to be considered in more detail. Heilbronner and Haber (Heilbronner and Haber 2014) found evidence for a cingulate bundle architecture involving four compartments, with some connections to limbic structures (such as the amygdala), and others not connected to such structures associated with affect/emotion and/or motivation. Therefore, it seems possible that white matter integrity of some but not all compartments of the CB is related to “cool” executive functioning, while white matter integrity of other compartments may be associated more strongly with “hot” executive functioning. A similar explanation may be suggested for the lack of significant associations between SLF white matter integrity and inhibitory control, the SLF also being a large bundle of white matter fibers, present in each hemisphere, and with potentially different functional significance in different segments. Such an explanation would corroborate with the fact that we found associations between whole brain white matter integrity and inhibitory control as well, as the contribution of the ATR WM-integrity (possibly together with the non-significant contributions of the CB and the SLF) to whole brain WM-integrity may be sufficient to render these correlations significant. However, in order to provide more definite answers future studies should segment further what are now considered unitary regions of interest, and include neuropsychological tasks that measure other executive functions such as working memory and cognitive flexibility and tasks that measure “hot” executive functions (see (Griffith-Lendering et al. 2012; Stephan C J Huijbregts et al. 2008; Rubia 2011)). One (further) reason for investigating this in more detail is that it has convincingly been shown that NF1-patients have extensive socio-emotional problems (Garg et al. 2013; S. C. Huijbregts et al. 2015) as well as impaired social information processing (Huijbregts et al. 2010a; Pride et al. 2014), and it seems reasonable to hypothesize that white matter integrity of tracts other than the ATR are more strongly associated with such problems.

Another important topic for future studies is to integrate what is known at neurobiological or cellular signaling level regarding NF1-pathophysiology and what has consistently been found at neuroanatomical level, and to investigate interrelations between different neuroanatomical abnormalities observed in NF1. Globally, we found mean and axial diffusivity to be negatively associated with inhibitory control. Mean diffusivity is a measure of the average molecular motion independent of any directionality and is affected by cellular size and integrity (Cercignani et al. 2001). Axial diffusivity was reported to be specifically sensitive to axonal degeneration (Alexander et al. 2007). In NF1, DTI-metrics have been related to T2-hyperintensities, although white matter abnormalities were also observed in lesion-free brain regions (Eastwood et al. 2001; Ferraz-Filho et al. 2011). The results of our study indicated that presence of T2-hyperintensities did not determine abnormalities of the microstructure in ATR in NF1-adolescents. Moreover, apart from some evidence showing associations between lesions at specific (subcortical) locations and cognitive outcomes, the hypothesis stating that T2-hyperintensities influence cognition lacks empirical support (S. C. Huijbregts et al. 2015; Hyman et al. 2007; Moore et al. 1996). Although results to date suggest only a relatively marginal role for T2-hyperintensities in both white matter microstructure and cognitive outcomes, whilst white matter microstructure itself seems to play a significant role in cognitive outcomes, it should be noted that the sample size of patients with and without T2-hyperintensities was limited, thus warranting further studies on this issue. Altogether, however, results of this and other studies suggest that concurrent pathological (e.g. neurobiological/cellular) processes affect white matter microstructure in NF1 patients.

As noted, the main limitation of this study was its sample size. However, the sample size is comparable to other imaging studies in NF1 (Karlsgodt et al. 2012; Pride et al. 2014; Violante et al. 2013). Perhaps more importantly, the strong involvement of white matter integrity of the ATR in inhibitory control was corroborated by high effect sizes (all above 0.8) and non-overlapping distributions when comparing NF1-patients and controls. Also, estimation of verbal and performance abilities and inhibitory control was not available for all subjects (with those data particularly not collected among healthy controls). Group differences in absolute numbers, which are similar to, or even larger than those observed in other studies (Huijbregts et al. 2010b; Rowbotham et al. 2009), indicating lower scores on IQ-subtests for NF1-patiens and more inhibitory control errors for NF1-patients than controls, may not always have reached significance due to limited power. Lastly, to underpin our speculations on different aspects of executive functioning possibly being associated with specific white matter tracts, tasks incorporating different executive function-dimensions have to be included in future studies.

It is concluded that, in NF1, microscopic white matter damage to the ATR is strongly associated with inhibitory control deficits. White matter integrity of the ATR may be predictive of other impairments in executive functioning characterizing NF1 as well, and thus represent an important target in therapeutic interventions. Although there is still much to be clarified, the single-gene disorder NF1 provides an extremely relevant model for studying associations between neurobiology, cell signaling, neuroanatomy and cognition/behavior as well.
